# Phytochemicals from Indian Ethnomedicines: Promising Prospects for the Management of Oxidative Stress and Cancer

**DOI:** 10.3390/antiox10101606

**Published:** 2021-10-13

**Authors:** Nishat Fatima, Syed Shabihe Raza Baqri, Ahmad Alsulimani, Sharmila Fagoonee, Petr Slama, Kavindra Kumar Kesari, Shubhadeep Roychoudhury, Shafiul Haque

**Affiliations:** 1 Department of Chemistry, Shia PG College, Lucknow 226003, India; nishatfatimalko@gmail.com; 2 Department of Zoology, Shia PG College, Lucknow 226003, India; ssrbaqri@gmail.com; 3 Medical Laboratory Technology Department, College of Applied Medical Sciences, Jazan University, Jazan 45142, Saudi Arabia; aalsulimani@jazanu.edu.sa; 4 Institute of Biostructure and Bioimaging (CNR), Molecular Biotechnology Center, 10124 Turin, Italy; sharmila.fagoonee@unito.it; 5 Department of Animal Morphology, Physiology and Genetics, Faculty of AgriSciences, Mendel University in Brno, 61300 Brno, Czech Republic; petr.slama@mendelu.cz; 6 Department of Applied Physics, School of Science, Aalto University, 00076 Espoo, Finland; kavindra.kesari@aalto.fi or; 7 Department of Life Science and Bioinformatics, Assam University, Silchar 788011, India; 8 Research and Scientific Studies Unit, College of Nursing and Allied Health Sciences, Jazan University, Jazan 45142, Saudi Arabia; 9 Faculty of Medicine, Bursa Uludağ University, Görükle Campus, Nilüfer, Bursa 16059, Turkey

**Keywords:** ethnomedicine, oxidative stress, ROS, cancer, phytochemicals

## Abstract

Oxygen is indispensable for most organisms on the earth because of its role in respiration. However, it is also associated with several unwanted effects which may sometimes prove fatal in the long run. Such effects are more evident in cells exposed to strong oxidants containing reactive oxygen species (ROS). The adverse outcomes of oxidative metabolism are referred to as oxidative stress, which is a staple theme in contemporary medical research. Oxidative stress leads to plasma membrane disruption through lipid peroxidation and has several other deleterious effects. A large body of literature suggests the involvement of ROS in cancer, ageing, and several other health hazards of the modern world. Plant-based cures for these conditions are desperately sought after as supposedly safer alternatives to mainstream medicines. Phytochemicals, which constitute a diverse group of plant-based substances with varying roles in oxidative reactions of the body, are implicated in the treatment of cancer, aging, and all other ROS-induced anomalies. This review presents a summary of important phytochemicals extracted from medicinal plants which are a part of Indian ethnomedicine and Ayurveda and describes their possible therapeutic significance.

## 1. Introduction

Living cells are subjected to constant wear and tear throughout their lives, and oxidative metabolism constitutes an important mechanism involved in this process. The cellular milieu is characterized by a tricky equilibrium between pro- and antioxidants, which dictates the overall health of organisms. Any imbalance in the relative abundance of key components of oxidative metabolism leads to oxidative stress which forms the basis of a variety of diseases. Failure of the body to deal with oxidative damage is presumed to be the reason for cell senescence, cancer, and many immunological disorders. Thus, understanding oxidative metabolism is an important prerequisite for devising suitable therapeutic strategies against such diseases.

Cancer is a global health problem because of its high prevalence and poor prognosis. It is an outcome of uncontrolled cell division which results from the loss of regulation over various cell cycle checkpoints. Cancer can involve almost every part of the body, a feature that accounts for the great diversity of known cancers. Many types of cancer have a clear genetic basis and arise from an altered expression of certain genes called oncogenes [1]. Important physiological roles are assigned to cellular oncogenes in a normal cell and most of them are involved in cell cycle regulation through their effects on molecular mechanisms of chromatin condensation, DNA damage, replication, transcription, and translation. Cellular oncogenes are homologous to viral oncogenes, which explains the viral origin of some well-known cancers. A different class of genes involved in cancer progression are tumor suppressor genes, such as p53 and retinoblastoma protein (Rb) that avert the development of cancer by inciting apoptosis in cancerous cells [2]. Any defect in tumor suppressor genes pushes the cancerous cell to escape apoptosis and continue proliferation leading to tumorigenesis [3].

Oxidative metabolism involving reactive oxygen species (ROS) has been shown to play a significant role in key cellular pathways of DNA repair and damage, cell cycle control, and apoptosis [4]. Since all these pathways are correlated with the incidence of cancer, it is obvious that oxidative stress has a huge bearing on cancer epidemiology. In fact, oxidative stress has been shown in causing cancer by inducing DNA damage and mutagenesis. As a result, antioxidant substances are seen as potential candidates in treating various types of cancer, and this has been a promising research area to explore further in cancer therapeutics. In some cases, the theoretical basis of anticarcinogenic effect of certain antioxidants is well known. In this connection, molecules involved in the oxidative metabolism of the cell (such as glutathione, superoxide dismutase - SOD, and peroxidase) appear to be the likely targets for managing cancer [4].

Extensive research has been performed on ways to treat cancer over the past few decades but an ultimate cure remains elusive. However, there has been a significant improvement in cancer prognosis due to the development of strategies such as radiotherapy, chemotherapy, and immunotherapy that aim at killing malignant cells through the use of radiation, powerful cytotoxic chemicals, or antibodies against tumor-specific antigens, respectively. A flip side of all these strategies is that they result in widespread cytotoxicity of healthy cells too and cause severe side effects such as hair loss and skin damage [5]. This has led to an intensified quest for alternative cures that are free of such side effects. Alternative medicine provides a ray of hope for a safe cancer cure exempt from undesirable side effects. Phytochemicals obtained from traditional herbs are known to have a wide chemical diversity, and several classes of such phytochemicals may have anticancer properties. In particular, chemicals such as terpenes, carotenoids, alkaloids, organosulfur compounds, and steroids are abundantly present in plants. Some of these have shown clinical efficacy in treating cancer [6,7]. We discuss these aspects in a detailed manner in the below mentioned sections.

## 2. Relation between Oxidative Stress and Cancer

Oxidative stress is described as a type of disturbance in the balance between the production of ROS [free radicals and non-free radical oxygen comprising chemical molecules such as hydrogen peroxide (H_2_O_2_), singlet oxygen, hydroxyl radical, and superoxide] and antioxidant defense [8]. Oxidative stress has numerous pro-tumorigenic effects such as genome instability, enhancing DNA mutation rate, or promoting DNA damage as well as cell proliferation. On the other hand, oxidative stress also exerts anti-tumorigenic activities and studies have shown that it is correlated with apoptosis and senescence - the two key mechanisms that neutralize cancer development [9]. Cancer cells exhibit enhanced ROS production that may increase cell proliferation. Undeniably, cancer initiation and its progression are correlated to oxidative stress-mediated DNA damage, increased DNA mutations or genome instability as well as cell proliferation [10]. In order to guard the organism against such detrimental pro-oxidants (endobiotic or xenobiotic), a systematically designed complex system of enzymatic antioxidants, for example, SOD, glutathione peroxidase (GPx), glutathione reductase, catalase and non-enzymatic antioxidants (which act by interrupting free radical chain reactions) such as glutathione (GSH), vitamins C, D and E come into play [11]. Under regular physiological situations, these protective antioxidant molecules are present in quantities adequate to tackle the physiological rate of free-radical production. It is also well known that any further load of free radicals can disturb the antioxidant balance (anti-free radical) and pro-oxidant (free radical) balance. This imbalance leads to oxidative stress, when it goes beyond the capability of the oxidation-reduction system of the body, gene mutation may result leading to many chronic diseases including cancer [12].

## 3. Indian Ethnomedicinal Plants as a Potential Source of Anticancer Phytochemicals

**Figure 1:**
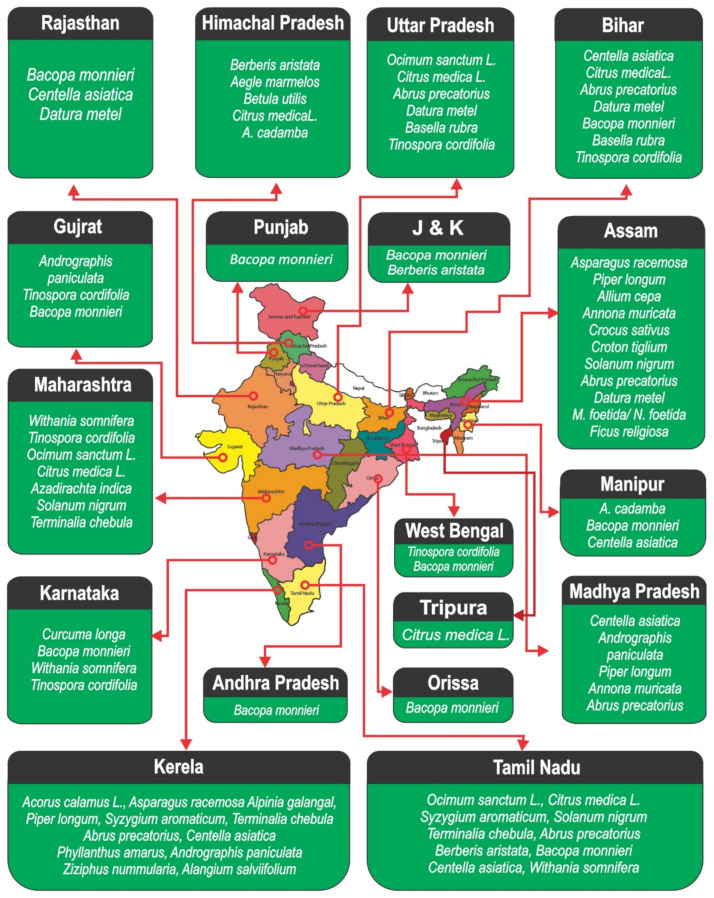
Geographical distribution of some prominent Indian ethnomedicinal plants.

Phytochemicals of remedial plants encompass a diverse chemical space for healthcare and management of common disorders. India is well known for its custom of traditional remedies and ethnopharmacology [13]. The Indian herbal medicines or the conventional medicaments have been derived from the prosperous customs of prehistoric civilizations and scientific inheritance. Herbal therapeutics, thus comprise a key stake of all the legitimate Indian organizations of medicine such as Ayurveda, Yoga, Unani, Siddha, and Homeopathy (AYUSH) [14]. These herbal remedies are used by more than 70% of the inhabitants for their primary health-related troubles [15]. It is believed that these plant-based herbal remedies are non-toxic against normal cells and are therefore well tolerated (or accepted) by the human body. This attribute is a great motivation to spark the interest of drug designers who are in the pursuit of cheap and safer alternatives to modern synthetic medicines. A brief list of some ayurvedic medicinal plants having potent anticancer activity and their main phytochemicals is provided in Table 1. A state-wise geographical distribution of some prominent Indian ethnomedicinal plants has been provided in Figure 1.

The currently used drug development program based on ayurvedic prescriptions has gained extensive reception in the existing healthcare system. Table 2 listed out several important Indian ethnomedicinal/Ayurvedic remedies and their uses in medical science. Although, validation of a combined therapeutic approach based on Ayurvedic prescriptions and modern medicine with better efficiency and safety is likely to be a big leap in overcoming some of the hurdles in the way of treating difficult disorders such as cancer [16].

Anticancer phytochemicals and their derived secondary metabolites present in leaf, root, flower, bark, and stem catalyze numerous pharmacological functions in human healthcare systems. Flavonoids, alkaloids, glycosides, phenolics, tannins, gums, oils, and resins are such representative compounds [17]. These phytochemicals or their modified derivatives have displayed significant anti-tumor potential.

There are four major classes of plant-derived or plant-based anti-cancer agents that are currently being used commercially: (1) the vinca alkaloids from *Catharanthus roseus* (vincristine, vinblastine, and vindesine), (2) the epipodophyllotoxins from *Podophyllum peltatum* (etoposide and teniposide), (3) the taxanes from the genus Taxus (docetaxel and paclitaxel) and (4) the camptothecin derivatives from *Camptotheca acuminata* (irinotecan and camptotecin) [18]. Moreover, the anti-cancer potential of a variety of plants is still being actively investigated and even some have displayed very encouraging results.

**Table 1:** A brief list of some ayurvedic medicinal plants having potent anticancer activity and their main phytochemicals.

Ayurvedic Name	Scientific Name	Family	Active/Antioxidant/Anticancer Phytochemicals	References
Amrita/Guduchi/Giloya	*Tinospora cordifolia*	*Menispermaceae*	Palmative, new clerodane furanoditerene glycoside, arabinogalactan, berberine, phenolic compounds, and epoxy cleodane diterpene	[19]
Amritavallari	*Basella rubra*	*Basellaceae*	p-Coumaric acid, caffeic acid, diosmetin	[20]
Amruta, Kalgur, or Narkya	*Mappia foetida/Nothapodytes foetida*	*Icacinaceae*	Camptothecin	[21]
Anlkol	*Alangium salviifolium*	*Cornaceae*	Deoxytubulosine, alangimarckine, dehydroprotoemetine, salviifosides A-C, kaempferol, salicin, and kaempferol 3-O-b-D-glucopyranoside	[22]
Ashwagandha	*Withania somnifera*	*Solanaceae*	Withanolides, sitoindosides	[23]
Ashwatha	*Ficus religiosa*	*Moraceae*	Quercetin, myricetin, kaempeferol, β-sitosteryl-D-glucoside	[24]
Bhukamtaka sukhsharanphala	*Ziziphus nummularia*	*Rhamnaceae*	Rutin, chlorogenic acid, quercetin, pyrogallol, mandelic acid, morin	[25]
Bhumyamalaki	*Phyllanthus amarus*	*Phyllanthaceae*	Nirtetralin (NIRT), niranthrin (NIRA), phyllanthin (PHYLLA), phyltetralin	[26]
Bhunimba and kalmegha	*Andrographis paniculata*	*Acanthaceae*	Andrographolide	[27]
Bhurjapatra	*Betula utilis*	*Betulaceae*	Betulinic acid	[28]
Bilva	*Aegle marmelos*	*Rutaceae*	Skimmianine, marmesin, imperatorin, cuminaldehyde, xanthotoxol	[29]
Brahmamanduki	*Centella asiatica*	*Acanthaceae*	Asiaticoside	[30]
Brahmi	*Bacopa monnieri*	*Scrophulariaceae*	Brahmine, herpestine, nicotinine, bacosides A and B, betulinic acid, wogonin, and oroxindin	[31,32]
Daruharidra	*Berberis aristata*	*Berberidaceae*	Berberine, berbamine, oxyberberine, aromoline, a protoberberine alkaloid karachine, palmatine, taxilamine, and oxycanthine	[33]
Dhatura	*Datura metel*	*Solanaceae*	Pterodontriol B, scopolamine, adenosine, disciferitriol, thymidine, dioscoroside D, and ilekudinoside C	[34]
Gunja	*Abrus precatorius*	*Leguminosae*	Abrin, Cycloartenol, Luteolin, Isoorientin, Trigonelline	[35]
Haridra	*Curcuma longa*	*Zingiberaceae*	Curcumin, Curcuminoid, Desmethoxycurcumin, Bisdemethoxycurcumin, Curdione, Bisacurone	[36]
Haritaki	*Terminalia chebula*	*Combretaceae*	Arjunglucoside I, chebulosides I and II, arjungenin, chebulin, 2,4-chebulyl-ß-D-glucopyranose, chebulic acid, chebulinic acid, terchebin	[37]
Kadamba, Vrattapuspa, Sisupala	*Anthocephalus cadamba*	*Rubiaceae*	Cadamine, isocadambine, isocadambine	[38]
Kakamachi	*Solanum nigrum*	*Solanaceae*	Solamargine, Solasonine, Solasodine, Solanidine	[39]
Kumbhinī	*Croton tiglium*	*Euphorbiaceae*	Corydine and salutaridine	[40]
Kumkuma/Ghusrun/Agneeshekhar	*Crocus sativus*	*Iridaceae*	Crocin, crocetin, picrocrocin, and safranal	[41]
Lakshamanap hala	*Annona muricata*	*Annonaceae*	Annonacin, Isoquinoline, Anonaine, Bullatacin, Annonamine, Gentisic acid	[42]
Lavanga	*Syzygium aromaticum*	*Myrtaceae*	Eugenol, Bicornin, Caryophyllene, Tellimagrandin II	[43]
Neem	*Azadirachta indica*	*Meliaceae*	Azadirachtin, Nimbin, Gedunin, epoxyazadiradione, Oleic acid	[44]
Nimbuka	*Citrus medica*	*Rutaceae*	Citronellal, Undecanal, Bisabolene	[45]
Palandu	*Allium cepa*	*Liliaceae*	Quercetin, Diallyl disulfide, Allicin	[46]
Pippali	*Piper longum*	*Piperaceae*	Piperine, Chavicine, Piperlongumine, Piperlonguminine	[47]
Rasna	*Alpinia galangal*	*Zingiberaceae*	Galangin, Kaempferide, Cadinene, fenchyl acetate	[48]
Rasona	*Allium sativum*	*Amaryllidaceae*	Alliin, allicin alliin, alliinase	[49]
Revandachini	*Rheum emodi*	*Polygonaceae*	Emodin, aloe-emodin, chrysophanol, physcion, rhein, emodin glycoside, and chrysophanol glycoside	[50]
Shatavari	*Asparagus racemosa*	*Liliaceae*	Shatavaroside A and B, shatavarins, filiasparoside C, immunoside, and schidigerasaponin	[51]
Sunthi	*Zingiber officinalis*	*Zingiberaceae*	Gingerol, Zingiberene, Shogaol, Paradol, Zingerone	[52]
Tulsi	*Ocimum sanctum*	*Labiatae*	Eugenol, Caryophyllene, Linalool, Methyl eugenol, Estragole, Carvacrol, Cadinene, Farnesene	[53]
Vacha, Ugargandha, Chhadgrantha	*Acorus calamus*	*Acoraceae*	Asarone, Cadinene, Methyl isoeugenol	[54]

**Table 2:** Some popular Indian ethnomedicinal/Ayurvedic remedies and their uses.

Ethnomedicinal/Ayurvedic Remedy	Plant Species	Description	Benefits and Medicinal Uses	References
Jatamansi	*Nardostachys jatamansi DC*	Ayurvedic Powder	Use as brain tonic that help to improve memory and brain functions by preventing cell damage due to its antioxidant property	[55]
Bhootkeshi	*Selinum vaginatum (Edgew) Cl*	Ayurvedic Powder	Sleep and mental disorders, high blood pressure	[56]
Haridra/Daruharidra	*Curcuma longa*	Ayurvedic Powder	Chronic anterior uveitis, rheumatoid arthritis, conjunctivitis, small pox, skin cancer, chicken pox, urinary tract infections, wound healing, and liver ailments	[57]
Drakshasava	*Vitis Vinifera, Woodfordia Fruticosa,* *Piper Cubeba, Cinnamomum Tamala,* *Mesua Ferrea, Syzygium Aromaticum* *Myristica Fragrans, Piper Nigrum,* *Plumbago Zeylanica, Piper Retrofractum* *Piper Longum, Vitex Negundo*	Ayurvedic rejuvenators	Weakness, lethargy and heat exhaustion, curing haemorrhoids and cardiac disorders	[58]
Kanchanar Guggulu	*Bauhinia variegata* L. *(BV),* *Zingiber officinale, Piper nigrum, Piper longum, Terminalia chebula, Terminalia bellerica, Embelica officinalis, Crataeva nurvala, Cinnamomum tamala, Elletaria cardemomum, Cinnamomum zeylanicum, Commiphora muku*	Ayurvedic Formulation	Hormonal imbalance, PCOS, hypothyroidism, and joint pains. weight loss, lipoma, tumor, cysts, cancer, goiter, fistula, boils and skin related diseases	[59]
Hingvastaka	*Zingiber officinale, Piper nigrum, Piper longum, Trachyspermum ammi, Carum carvi, Cuminum* *Cyminum, Ferula asafoetida*	Ayurvedic churna	Indigestion, Anorexia and all Vata Disorders, relieve flatulence	[60]
Triphala	*Phyllanthus emblica,Terminalia bellirica, Terminalia chebula*	Polyherbal Ayurvedic medicine	Indigestion, weight loss,Reducing inflammation, regulate blood sugar levels, Lower cholesterol, reduce tumors, prevents cancer, inhibit HIV	[61]
Aswagandharishtam	*Withania somnifera, Rubia cordifolia,* *Chlorophytum tuberosum, Terminalia chebula* *Curcuma longa, Berberis aristata, Glycyrrhiza glabra, Pluchea lanceolata, Pueraria tuberose* *Terminalia arjuna, Cyperus rotundus, Ipomoea turpethum, Hemidesmus indicus, Santalum album* *Acorus calamus, Plumbago indica*	Liquid Ayurveda medicine	Anti-anxiety, anti-stress, antidepressant	[62]
Kasisadi Tailam	*Leucaena Leucocephala, Ficus Religiosa, Dry Ginger, and Plumbago Zeylanica*	Polyherbal Ayurvedic oil	External application on corns, piles and warts	[63]
Pusyanuga curna	*Cissampelaos pareira, Syzygium cumini, Mangifera indica, Bergenia lingulata, Berberis aristata, Ambastha (Patha)- Cissampelos pareira, Mochrasa- Salmalia malabarica, Mimosa pudica, Holarrhena antidysentrica, Crocus sativus, Aconitum heterophylum, Aegle marmelos, Cyperus rotundus, Symplocos racemosa, Ochre or Haematite, Ailanthus excels, Piper nigrum, Zingiber officinale, Vitis vinifera, Pterocarpus santalinus, Myrica esculenta, Holarrhena antidysentrica, Hemidesmus indicus, Woodfordia fruticosa, Glycyrrhiza glabra, Terminalia arjuna*	Herbo-mineral formulation	Conditions involving menstrual irregularities such as menorrhagia,metrorrhagia, dysmenorrhoea and endometriosis, piles, diarrhoea, bloody stools and different types of discharges from vaginal tract	[64]
Lashuna taila	*Allium Sativum linn, Sesamum indicum linn*	Ayurvedic oil	Fungal infections, warts, and corns, hair loss and thrush	[65]

## 4. Antioxidant Phytochemicals in the Management of Cancer

It is well established that the incidence of cancer and its progression has been associated with oxidative stress owing to the role played by oxidative agents in mutagenesis and induction of DNA damage which lead to cell proliferation and genome instability. This gives credence to the speculation that antioxidant agents could intercept carcinogenesis [66]. A plethora of studies has reported that phytochemicals from ethnomedicinal plants can yield numerous bioactive compounds with promising anti-cancer potential. Besides, in addition to their renowned antioxidant properties, several phytochemicals target epigenetic processes participating in cancer progression by modulating oxidative stress [67,68]. In treating cancer, an efficient strategy is to induce senescence and cytostasis by using cytotoxic agents. Several anticancer polyphenols arrest cellular growth via the induction of ROS-dependent premature senescence (RDPS), and are contemplated as promising antitumor therapeutic options [69]. In the light of this mechanism, pro-oxidant therapy which involves drugs that trigger oxidation-induced cell death has been emerging as an effective strategy for treating cancer [70].

**Figure 2:**
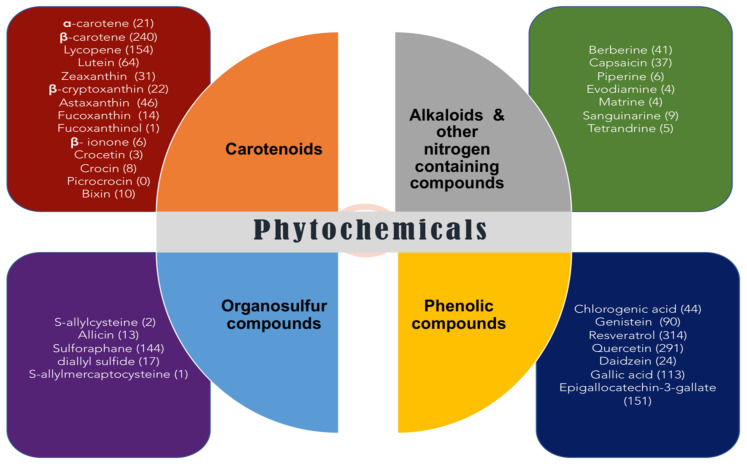
Illustration of the most reviewed antioxidant phytochemicals associated with oxidative stress and cancer. The structure of published literature is based on the citations in PubMed (April 2021). The numbers of published manuscripts are provided in parentheses along with the name of each phytochemical and “oxidative stress and cancer”, within title/abstract, is stated.

Several recent epidemiological researches have demonstrated that nutritional antioxidant phytochemicals such as phenolic compounds, carotenoids, flavonoids, and alkaloids can act as defensive agents against many chronic diseases including cancer [71]. In the recent past, numerous studies have been conducted to elucidate the mechanisms that impart anticarcinogenic properties to phytochemicals, such as vitamin C, vitamin E, carotenoids, flavonoids, and phenolic acids [72]. Curcumin, which is a bright yellow polyphenolic phytochemical produced by *Curcuma longa* (Turmeric) plants and is widely used in Indian ethnomedicine, is in fact a strong antioxidant agent. It is an exceptionally potent, lipid-soluble compound owing its healing properties to its pro-oxidant/antioxidant effects since the generation of ROS by curcuminoids and curcumin correlates with their apoptotic activity on cancer cells [73]. Antioxidant or pro-oxidant capacity of any prospective candidate drug against cancer thus appears heavily dependent on the concentrations of active phytochemicals in it. A number of studies using different cell models have highlighted the pro-oxidative properties of polyphenols, which are also known as antioxidants, such as genistein, quercetin, catechins, epicatechin, and resveratrol [74,75]. Figure 2 presents the structure of published articles indexed in PubMed online database pertaining to the most studied antioxidant phytochemicals associated with oxidative stress and cancer.

Quercetin can be used for averting cancer via the modulation of oxidative stress factors and several antioxidant enzymes for preventing the progression of a variety of cancers, such as lung, liver, prostate, colon, breast, and cervical cancers [75,76]. In an in vivo research study performed on rats by Sharmila et al., the antioxidant activity of quercetin was matched with that of a carcinogen and testosterone by histological evaluation and measuring oxidative stress markers, such as lipid peroxidation (LPO), H_2_O_2_ and reduced GSH. They observed that carcinogen and testosterone-treated rats had higher levels of LPO and H_2_O_2_ and lower levels of GSH compared with rats with quercetin treatment. They also reported that quercetin improved the levels of antioxidant enzymes and apoptotic proteins in animals having prostate cancer. Additionally, the authors observed that quercetin synchronized the expression of androgen receptors (AR), insulin-like growth factor receptor 1 (IGFR1), protein kinase B (AKT), cell proliferation factors, and anti-apoptotic proteins which are usually elevated in cancer [77]. Quercetin also trims down the overproduction of ROS, inhibits the expression of tumor necrosis factor (TNF)-α gene, corrects the damage inflicted by trauma and averts myocardial cell injury caused due to Ca^2+^ overload [78]. Thus, quercetin can efficiently prevent injuries caused by oxidative stress [79].

Furthermore, silymarin from milk thistle, green tea polyphenols, and proanthocyanidins from grape seeds all have the capability to defend the skin from the unfavourable effects of UV radiation including skin cancers. The protection against UV damage was achieved mainly through four mechanisms, namely inhibition of DNA damage, induction of inflammation, suppression of immune responses, and oxidative stress [80].

Resveratrol is a stilbene-type aromatic phytoalexin, mainly available in red wine, grape skin, peanuts, berries, purple grape juice, turmeric, and other dietary foodstuffs. Resveratrol has shown potential anticancer activity primarily because of induction of apoptosis through several pathways, all leading to a reduction in tumor initiation, promotion, and progression, and alteration in gene expression [81]. Lately, it is reported that the antioxidant potential of hydroxyl stilbenes (trans- and cis-resveratrol and their hydroxylated analogs) is associated with the regulation of eicosanoid synthesis, and the basic stilbene structure of two benzene rings bonded by a central ethylene is chiefly accountable for its properties on Caco-2 cell growth, DNA synthesis, and cell cycle independently of redox state and modulation of eicosanoid synthesis [82].

Lycopene is a non-provitamin A carotenoid which has shown potential anticancer activity against advanced and aggressive conditions of prostate cancer [83]. Lycopene and its auto-oxidant products may stimulate apoptosis in HL-60 cells. It is involved in modulating Bcl-2, thereby influencing apoptosis, which may explain the observed antioxidant properties of lycopene. It also restrains carcinogenesis by saving indispensable biomolecules such as proteins, lipids, LDL, and DNA [84]. Besides, lycopene also possesses the quenching capability against singlet oxygen that can be accredited to its conjugated double bonds [85].

## 5. Classification of Dietary Antioxidant Phytochemicals in the Management of Oxidative Stress and Cancer

### 5.1. Phenolic Compounds

Phenolic compounds represent one of the largest and most extensive groups of secondary metabolites in the plant kingdom and are characterized by an aromatic ring holding one or more hydroxyl groups. More than 8000 natural phenolic compounds have been identified which are divided into different subgroups such as phenolic acids, tannins, flavonoids, coumarins, quinones, lignans, curcuminoids, and stilbens [86].

**Figure 3:**
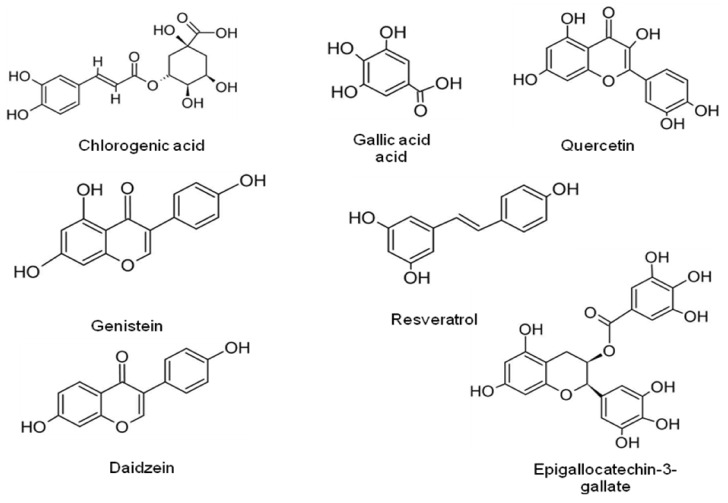
Chemical structures of some lead phenolic phytochemicals.

Flavonoids are further classified as flavonols, flavones, flavanones, isoflavonoids, and anthocyanidins. Polyphenols are explored as promising medicinal agents for the treatment of various diseases including ulcer, bacterial infections, hypertension, allergies, hypercholesterolemia, and vascular fragility, especially in various types of cancer. Table 3 lists the anticancer activity of some recently reported potent antioxidant phenolic phytochemicals, whereas Figure 3 shows the chemical structures of some lead phenolic phytochemicals.

There is a popular faith that nutritional polyphenols have anti-cancer properties because of their anti-oxidative characteristics. Their conjugated structures give rise to wonderful electron delocalization characteristics that bestow the ability to quench free radicals and react with a large number of ROS, including singlet oxygen, superoxide radical, nitric oxide, peroxyl radical, hydroxyl radical, peroxynitrite and nitrogen dioxide. The occurrence of numerous hydroxyl groups in their structures makes polyphenols exceptional hydrogen bond donors. Their ability to bind hydrogen is supposed to be responsible for their elevated affinity for nucleic acids and proteins. Polyphenols have a molecular basis to induce cancer cell death by the down-regulation of oncogenic survival kinases such as PI3K and Akt, D-type cyclins, cyclin-dependent kinases (CDKs), cell proliferation regulators that include Erk1/2, angiogenic factors such as VEGF, FGFR1, and MIC-1 and transcription factors such as NF-kβ, NRF2, and STATs etc. [87].

Among all the polyphenols, flavonoids are the most abundant in nature, having a basic skeleton of phenylbenzopyrone consisting of two aromatic rings. They can occur in nature in both free and conjugated forms. They have a wide range of anti-cancer and anti-mutagenic properties. However, whether a particular polyphenol is antimutagenic or not solely depends upon its chemical structure, the concerned gene, and the mutagenic factor (alcohol, ultraviolet radiation, tobacco consumption etc.). 

Resveratrol (RE; 3,4′,5 trihydoxystilbene) is a stilbenoid natural polyphenol, found in many Indian ethnomedicinal plants such as Drakshasava (*Vitis vinifera* L.), Dadima (*Punica granatum* Linn. fruits), and Shahtoot (*Morus indica*) [88]. It demonstrated potential inhibitory activity in different cancer types, such as for the initiation, promotion, and progression stages.

The apoptotic signals are originated through two main pathways: (i) the intrinsic pathway and (ii) the extrinsic pathway. The intrinsic pathway operates by stimulating the mitochondrial membrane to hinder the expression of anti-apoptotic proteins Bcl-2 and Bcl-Xl [89]. Curcumin perturbs the balance in the mitochondrial membrane potential, leading to enhanced suppression of the Bcl-xL protein [90]. 

Ginger has served as a principal drug in Ayurveda, Unani, Siddha, Chinese, Homeopathy, and Tibetan traditional medicines for more than 2000 years [91]. It contains a variety of active phenolic compounds, e.g., [6]-gingerol, [8]-gingerol, [10]-gingerol, and [6]-shogaol. Among these, [6]-gingerol is one of the most significant pungent components of ginger and was found to have varied pharmacological actions, such as antioxidant, antiemetic, cardiotonic, anti-tumor, anti-inflammatory, and anti-platelet properties [92]. de Lima et al. (2018) found that ginger extract and [6]-gingerol exerted their action through imperative mediators and pathways of cell signaling, including Bax/Bcl2, Nrf2, p38/MAPK, p65/NF-κB, ERK1/2, SAPK/JNK, ROS/NF-κB/COX-2, TNF-α, caspases-3, -9, and p53 [93].

The vitamin E family, tocopherols (α-, β-, γ-, δ-), and tocotrienols (α-, β-, γ-, δ-) are an important group of fat-soluble phenolic compounds having strong antioxidant and anticancer properties. The major Indian ethnomedicinal dietary sources of tocopherols (vitamin E) are Vatada (*Prunus dulcis*), Surajmukhi seeds (*Helianthus annuus*)*,* and Gehu jawara (wheatgrass oil). There are several reports demonstrating that tocopherols mixture rich in γ-tocopherol inhibits lung, mammary, prostate, and colon tumorigenesis in animal models [94]. It is also evident by numerous in vitro and in vivo reports that γ-tocopherol, δ-tocopherol, and vitamin E metabolite 13’-carboxychromanol have potent anti-cancer activities via modulating key signaling and several mediators that regulate tumor progression and cell death for example PI3K, NF-κB, STAT3, sphingolipid and eicosanoids metabolism [95].

Flavonoid riboflavin (vitamin B2) is a water-soluble vitamin found in milk, dairy products, green leafy vegetables, fortified cereals, and grain products. This vitamin is necessary for normal cell growth and function, is required to process fats and amino acids, and helps convert carbohydrates into fuel. Along with several medicinal properties, this vitamin also has antioxidant nature and can protect the body against oxidative stress, reperfusion oxidative injury, and especially LPO [96].

**Table 3:** Anti-cancer activity of some potent antioxidant phenolic phytochemicals (recent data).

Bioactive Molecules	Indian Ethnomedicinal Plant Source	Chemical Classification	Associated Condition	Cell Line Tested	Biological Approach (In Vitro/In Vivo)	Mechanism of Action	References
Chlorogenic acid	Jjatamansi (*Nardostachys jatamansi*)	Ester of caffeic acid and (−)-quinic acid	Osteosarcoma	U2OS, Saos-2, and MG-63 OS	In vitro	Activates extracellular-signal-regulated kinase1/2 (ERK1/2)	[97]
Epigallocatechin-3-gallate	Syamaparni (*Camellia sinensis*)	Ester of epigallocatechin and gallic acid	Oral squamous cell carcinoma	KBV200	In vivo	Inhibition of angiogenesis via VEGF down-regulation	[98]
Genistein	Rajapatha (*Stephania glabra*)	Isoflavones	Kidney cancer		In vitro	Induces apoptosis and inhibit the proliferation via regulating CDKN2a methylation	[99]
Resveratrol	Drakshasava (*Vitis vinifera* L.)	Stilbenoid	Renal cell carcinoma		In vitro/in vivo	Depressing activity of NLRP3, and NLRP3	[100]
Quercetin	Tulsi (*Ocimum sanctum*)	Flavonol	Malignant melanoma	B16	In vitro	Reduced the proportion of cells in the S and G2/M stages of the cell cycle	[101]
Daidzein	Vidari (*Pueraria tuberosa*)	Isoflavones	Colorectal cancer		In vivo	Lessened the protein expression of p-ERK/ERK and p-AKT/AKT	[102]
Gallic acid	Amla (*Emblica officinalis* )	Phenolic acid	Prostate cancer	PC-3	In vitro/in vivo	Inhibits HDAC1 and 2 expression	[103]

### 5.2. Alkaloids

Alkaloids are amongst the highly copious plant secondary metabolites. They create a large conglomerate of fundamental heterocyclic nitrogen encompassing natural phytochemicals that are usually produced by plants as toxic substances [17]. Out of 27,000 diverse alkaloids known, nearly 17,000 have shown varied pharmacological properties comprising anticancer activities [104]. Among the many pharmacological features of alkaloids are their prominent antioxidant properties which make them capable of averting a range of degenerative ailments either through their binding to catalysts involved in diverse oxidation processes happening within an individual’s body or through capturing free radicals.

**Figure 4:**
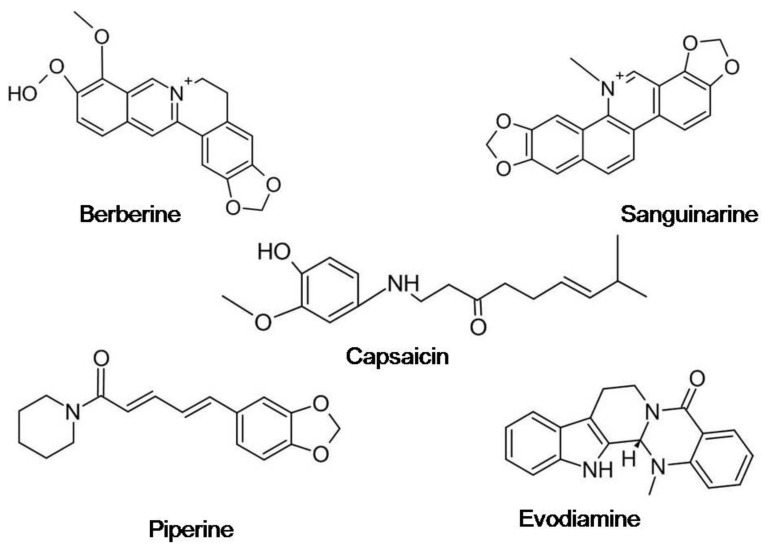
Chemical structures of some lead alkaloids.

Alkaloids can be classified into several groups such as pyrrolizidines, quinolizidines, pyrrolidines, indoles, piperidines, tropanes, purines, isoquinolines, and imidazoles. Among these groups, the leading anticancer alkaloids isolated from the plants are compounds such as taxol, vinblastine, vincristine, topotecan, vinflunine, and camptothecins while other important isolated alkaloids include harringtonine, rohitukine, thalicarpine, acronycine, usambarensine, matrines, and ellipticine. Several studies have proved the efficacy of plant-based alkaloids in oncogenesis inhibition. Alkaloids have been shown to modulate key signaling pathways participating in proliferation, metastasis, and cell cycle regulation, and these very properties form the basis of their use as the principal components of numerous clinical anti-cancer agents. Table 4 lists the anticancer activity of some recently reported potent antioxidant alkaloids, whereas Figure 4 shows the chemical structures of some lead alkaloids.

Vinca alkaloids, colchicine, and paclitaxel are amongst the most primitive plant-derived anticarcinogenic compounds. These are microtubule-targeting agents which can be used to abort spindle formation during the cell division of rapidly dividing cancer cells. Thus, they are of clinical significance against many types of cancer such as ovarian, breast, prostate, and non-small cell lung cancer. 

Berberine is an isoquinoline derivative extracted from *Berberis aristata* also known as “Daruharidra” in Ayurveda. It is a wild shrub found at altitudes of 5000 feet and above in the Nepal and Himalayas region [105]. It is an isoquinoline derivative extracted from *Coptis chinensis* [Family; Berberidaceae], which possesses diverse pharmacological actions. It is equipped with anti-bacterial, anti-diabetic, anti-inflammatory, and anti-cancer properties and is also beneficial for the cardiovascular system [106].

Quite recently, Wang ZC et al. synthesized some novel berberine derivatives with di-substituents on positions C9 and C13 and assessed their anticarcinogenic activity against breast cancer cell line (MDA-MB-231), human prostate cancer cell lines (PC3 and DU145), and human colon cancer cell lines (HT29 and HCT116). Out of these, a specific compound designated as 18e showed the highest cytotoxicity against PC3 cells having an IC_50_ value of 0.19 μM. Additional studies revealed that 18e can arrest the cell cycle at the G1 phase and can extensively stop tumor cell colony formation and migration even at very low concentrations. Remarkably, 18e could notably bring about cytoplasmic vacuolation, suggestive of a different mechanistic action from berberine. It is well evidenced that potential molecular targets, as well as mechanistic action of berberine, are rather complex. It interacts with RNA or DNA to form a berberine-RNA or a berberine-DNA complex, respectively.

Rohitukine is a chromone alkaloid, that was initially isolated from the leaves and stems of *Amoora rohituka* widely used in the Ayurvedic system of medicines [107]. It has been shown to possess many important biological functions including anti-inflammatory, anti-fertility, and immunomodulatory effects [108]. The unique chemical structure of rohitukine presents a framework for derivatization and chemical synthesis of some novel molecules [109]. P-276-00 and flavopiridol are two rohitukine analogs presently evaluated in the advanced phase II clinical trials for a potential anticancer drug [110]. Flavopiridol demonstrated efficient action against most cancer cell lines and tumorous growth suppression in animals [111]. It is a pan-CDK inhibitor that arrests the cell cycle in G1/S or G2/M phase and has been used in the treatment of chronic lymphocytic leukemia [112]. In a recent study, it has been observed that rohitukine induces cytotoxic effects in lung cancer (A549) cells and stimulates the production of ROS following the exposure of 24 hrs. It was also found that rohitukine activated apoptosis in A549 cell line through the upregulation of p53 and caspase 9 and the downregulation of Bcl-2 protein [113].

**Table 4:** Anticancer activity of some potent antioxidant alkaloids (recent data).

Bioactive Molecules	Indian Ethnomedicinal Plant Source	Chemical Classification	Associated Condition	Cell Line Tested	Biological Approach (In Vitro/In Vivo)	Mechanism of Action	References
Berberine	Daruharidra (*Beberis aristata*)	Isoquinoline alkaloid	Colon cancer		In vitro	Targets SCAP/SREBP-1 pathway driving lipogenesis	[114]
			Chronic lymphocytic leukemia		Clinical trial/human patient	Induces apoptosis by decreasing ROR1, Bcl-2, and mir-21	[115]
Capsaicin	Twak (*Cinnamomum verum*)	Capsaicinoids	Prostate cancer	PC-3, DU145	In vitro	Suppressionof prostate cancer stem cells via inhibition of Wnt/β-catenin pathway	[116]
Piperine	Pipali (*Piper nigrum)*	N-acylpiperidine	Human melanoma cells	A375P, A375SM	In vitro and in vivo	Increased the expression of BCL2-associated X, apoptosis regulator (BAX), cleaved poly (ADP-ribose) polymerase, cleaved caspase-9, phospho-c-Jun N-terminal kinase and phospho-p38, and reduced that of B-cell lymphoma 2 (BCL2)	[117]
Sanguinarine	Svarnakshiri (*Argemone mexicana*)	Benzophenanthridine alkaloid	Multiple myeloma	U266, IM9, MM1S, and RPMI-8226	In vitro	Induces mitochondrial/caspase-dependent apoptosis, produces oxidative stress, and suppresses proliferation cancer cell lines	[118]
Tetrandrine	Patha (*Cissampelos pareira*)	Bis-benzylisoquinoline alkaloid	Breast cancer	MDA-MB-231	In vivo	TET-upregulated Caspase-3, Bid, Bax, and, downregulated by Bcl-2, Survivin, and PARP	[119,120]

Pain is an important point of consideration in dealing with cancer patients especially when its severity increases too much in advanced stages of the disease. There are two types of pain depending on its origin. The nociceptive pain which is sensed by nociceptors (pain receptors) is of peripheral origin, arising in bodily organs due to the invasive growth of the tumor or due to invasive surgical procedures. The second type of pain which is neuropathic and is either central or peripheral in origin occurs when the growing tumor in its advanced stages invades into the nervous tissue. Very often, the pain is iatrogenic and is caused by the drugs used in the treatment of cancer. Vinca alkaloids used as chemotherapeutic agents have been found to cause such iatrogenic pain. One estimate suggests that pain is not properly managed in more than half of cancer patients and a quarter of all cancer patients eventually die of pain. There exists a three-step guideline from the WHO to manage pain in cancer patients and it recommends the use of opiate alkaloids in the second and third stages of cancer. However, the use of opiate drugs is discouraged because of the fear of dependency and addiction, yet a study has found that such apprehensions are not true and addiction has not been reported in cancer patients taking opiates [121]. The standard strategy of using opiates as analgesics involves the use of alkaloids such as codeine in mild pain and morphine or other members of the class in later stages of severe pain [122]. It has long been known that morphine and other opiate alkaloids work by binding to μ-opiate receptors in the central nervous system to suppress pain [123].

### 5.3. Organosulfur Compounds 

**Figure 5:**
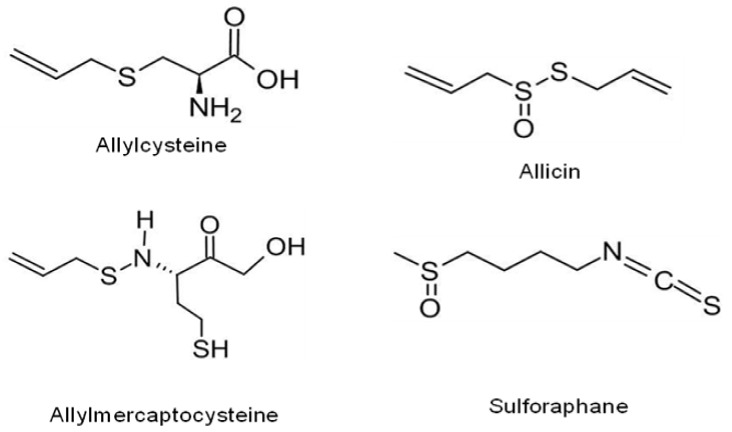
Chemical structures of some lead organosulfur phytochemicals.

Organosulfur compounds (OSCs) are organic molecules that contain sulfur principally responsible for the characteristic odour and flavour of onion and garlic. They are also abundant in cruciferous vegetables such as cabbage and broccoli [124]. There are two major groups of natural resources that contain OSCs with unique properties. Onion, garlic, shallot chives, and leeks are famous representatives of the *Allium* genus that have S-alk(en)yl-l-cysteine sulfoxides [125]. Cauliflower, cabbage, and kale are representatives of the genus *Brassica*, while rucola belongs to the genus *Eruca* of cruciferous family that contain S-methyl cysteine-l-sulfoxide [126]. Earlier research studies have revealed the anti-cancer mechanisms of extracts, preparations, and organosulfur products of alliaceous vegetables. These mechanisms presumably include their antimicrobial activity, redox modification, and reduced bioactivation of cancer-causing agents. Hence, *Allium* vegetables and their active phytochemicals have assumed significance in efficiently regulating the biological processing of carcinogenesis so as to alter or reduce the hazard of cancer. OSCs inhibit the development of cancer cells by several pathways including inhibition of metabolism, inhibition of angiogenesis, and cell cycle arrest. These compounds are classified into water-soluble OSCs such as S-Allyl Mercaptocysteine and S-Allyl Cysteine and oil-soluble OSCs, such as diallyl disulfide, diallyl sulfide, diallyl trisulfide, ajoene, and dithiins [127]. Numerous organosulfur compounds are declared to have potent antioxidant capacity. Their potential to scavenge ROS plays conceivably a significant role in their anti-senescence activity in vitro. Table 5 reports the anticancer activity of some recently reported potent antioxidant organosulfur phytochemicals and Figure 5 shows chemical structures of some lead organosulfur phytochemicals.

Alliin, allicin, S-allylcysteine, and S-allylmercaptocysteine are some major phytochemicals present in garlic, which is commonly known as ‘Lasuna’ in Sanskrit, considered to be one of the best Ayurvedic medicine and termed as Mahaaushadha [128]. Recently, Rosas-González et al. appraised the effects of alliin and allicin on cell death and senescence, and their senolytic potential in MCF-7 (luminal A) and HCC-70 (triple-negative breast cancer cells). Their results revealed that allicin has anti-proliferative, anti-clonogenic, and senolytic properties. In addition, allicin reduced cell viability and induced apoptosis by triggering the loss of ΔΨm, caspase-3, caspase-8, and caspase-9 activation, downregulation of BCL-XL expression as well as upregulation of NOXA, BAK, and P21. On the other hand, alliin endorsed clonogenicity, induced senescence, and did not demonstrate pro-apoptotic effects in breast cancer cells [129]. Allicin also suppressed proliferation and induced glioma cell apoptosis in vitro by intrinsic mitochondrial and extrinsic Fas/FasL mediated pathways [130].

Allicin is extremely unstable and is easily transformed into lipid-soluble sulfides such as diallyl sulfide (DAS), diallyl disulfide (DADS), and diallyl trisulfide (DATS) [131]. DAS, DADS, and DATS were revealed to play a significant role in inducing histone acetylation thereby resulting in the suppression of the oncogenic protein expression in human glioblastoma cells [132]. It has also been shown that DATS and DAS have the ability to induce apoptosis in the carcinogen-induced two-stage mouse model of skin tumour via targeting of multiple cancer signaling pathways [133].

Some garlic-based water-soluble phytochemicals such as S-allylmercaptocysteine (SAMC) and S-allyl cysteine (SAC) also exhibited potent anticancer activity [134]. SAC could also exhibit stronger antioxidant properties than fresh garlic, and has powerful anti-cancer activity not only at the early stage but also at the late stages of the ailment.

**Table 5:** Anticancer activity of some potent antioxidant organsulfur phytochemicals (recent data).

Bioactive Molecules	Indian Ethnomedicinal Plant Source	Chemical Classification	Associated Condition	Cell Line Tested	Biological Approach (In Vitro/In Vivo)	Mechanism of Action	References
S-allylcysteine	Lasuna (Fresh garlic)	Derivative of the amino acid cysteine	Lung cancer	A549	In vitro/in silico	Reduced expression of PD-L1 and HIF-1α, showed efficient docking with PD-L1 immune checkpoint target	[135]
Allicin	Lasuna (Crushed or chopped garlic)	Sulfoxide	Skin cancer/melanoma	A375	In vitro	Reduction of MMP-9 mRNA expression	[136]
			Cholangiocarcinoma	MCF-7, HCC-70	In vitro/in vivo	Overpowers cell proliferation and invasion through STAT3 signaling	[137]
S-allylmercaptocysteine	Lasuna (Aged garlic)	Thioallyl compound	Lung cancer	A549	In vitro	Suppression of cell proliferation, regulation of cell cycle, attenuation of ROS formation, inhibition of DNA damage, increase of SOD activity and inhibition of nuclear factor-kappa B (NF-κB) activity	[138]
Sulforaphane	Shatavari (*Asparagus racemosus*)	Isothiocyanate	Small-cell lung cancer	SCLC cell lines NCI-H69, NCI-H69AR and NCI-H82	In vitro	Induced cell death was mediated via ferroptosis and Inhibition of the mRNA and protein expression levels	[139]

### 5.4. Carotenoids

Carotenoids are tetraterpenes mainly synthesized in plants with a parent skeleton of hydrocarbon, which consists of C_40_H_56_ with definite rotation in single and double bonds. As of now, more than seven hundred carotenoids have been identified and can be categorized as carotenes or xanthophylls [140]. Furthermore, carotenoids can be oxidatively sliced by dioxygenase derivatives, called apocarotenoids [141].

A polyene backbone consisting of a sequence of conjugated C=C bonds forms the “core” structural element of carotenoids. This special feature is mainly accredited for both, their pigmenting properties and the capability of many of these phytochemicals to interrelate with singlet oxygen and free radicals, and consequently act as efficient antioxidants [142]. Carotenoids are the primary line protection mechanism that extinguishes oxygen either physically by energy relocation mechanism or chemically through direct reaction with the radicals [143]. Several carotenoids work as powerful antioxidants and anticancer agents. Various epidemiologic and investigational studies have reported that eating of carotenoids is inversely proportional with the incidence of cancer [144,145].

Nutritionally, carotenoids can be divided into two groups, pro-vitamin A and non-provitamin A. Out of thousands of known phytochemicals, only three of them (α-carotene, β-carotene, and β-cryptoxanthin) act as pro-vitamin A. They can be converted in vivo into vitamin A (retinol). Major dietary sources of non-provitamin A are lutein, lycopene, and zeaxanthin [146]. There are several epidemiologic studies suggesting that carotenoids reduced the hazard of different chronic ailments, especially cancers of gastrointestinal tract, pancreas, lung, and breast with dietary consumption of different carotenoids [147]. On the other hand, randomized clinical trials have not shown any reliable decrease in the frequency of cancers or cancer mortality [144]. Worst of all, in a study on asbestos workers and smokers, an elevated risk of lung cancer had been reported, whether they were given high doses of beta-carotene only or in combination with other antioxidants [148].

**Figure 6:**
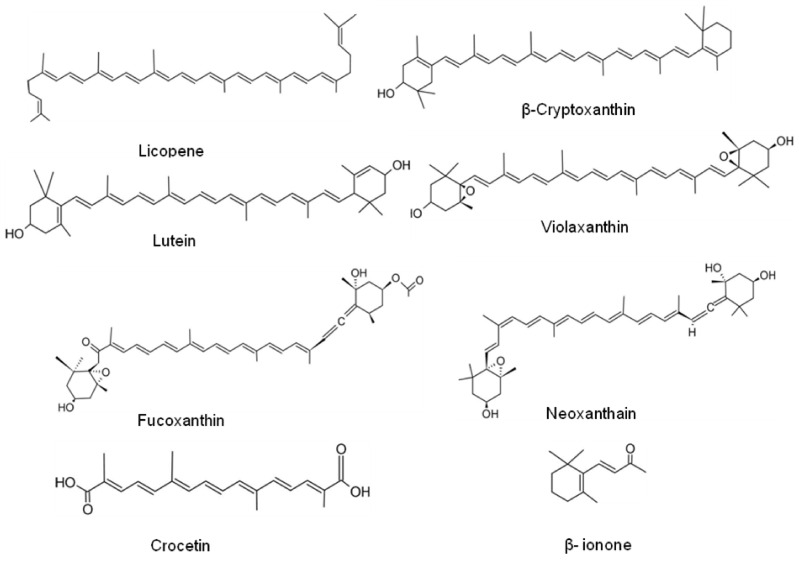
Chemical structures of some lead carotenoids.

Carotenoids such as β-carotene, α-carotene, lycopene, β-cryptoxanthin, violaxanthin, lutein, fucoxanthin, neoxanthain, canthaxanthin, zeaxanthin, astaxanthin, and siphonaxanthin have been proven for bearing anticancer activity in different cancer cells such as breast, colon, prostate, cervix, leukemia, and liver [149,150]. The proposed mechanisms of cancer chemoprevention using carotenoids probably involve modulations in pathways leading to cell development or cell demise. These mechanisms comprise hormone and growth factor signaling, immune modulation, regulatory mechanisms of cell cycle development, cell differentiation, and apoptosis [151]. The anticancer activities of some recently reported potent antioxidant carotenoids are listed in Table 6, whereas, chemical structures of some leading carotenoids are given in Figure 6.

## 6. Conclusions and Future Prospects

The importance of oxidative metabolism in the context of health and disease cannot be overlooked. All the major cellular and molecular pathways rely on redox reactions involving oxidizing and reducing equivalents such as NADH.H^+^, NADPH.H^+^, and FADH_2_. For their normal functioning cells need to maintain an equilibrium between pro-oxidants and antioxidants, failing which they stand to face consequences of oxidative stress. Overproduction of ROS is detrimental and interferes with cellular processes of DNA synthesis and repair, cell-cycle control, protein synthesis, and regulation of gene expression. These are critical mechanisms that decide the fate of a cell by influencing its survival and longevity. A cell exposed to oxidative stress is likely to undergo gene defects, apoptosis, and premature senescence. Therefore, cells are naturally equipped with mechanisms to cope with oxidative stress. In humans, glutathione, SOD, vitamin C (ascorbate), and vitamin E (tocopherol) are specifically concerned with defense of the body against oxidative stress. Herbal and ethnomedicinal drugs are usually effective against stress owing to the presence of some bioactive compounds.

The results of studies presented here are quite reassuring considering the enormous range of compounds that can be obtained from the herbs and their therapeutic potential. A number of findings presented in this review point to the fact that herbal extracts are particularly rich in compounds implicated in oxidative metabolism. In the case of many classes of phytochemicals, the basis of their therapeutic effects is known at the cellular and molecular level. However, there are many ethnomedicinal prescriptions which are considered effective against certain diseases but the exact nature of their effects is not clearly understood. In such cases, it is advisable to exercise caution because the claim that herbal preparations are always safe is misleading in nature. Toxicity associated with many herbal medicines has been reported in numerous studies and the adverse effects of herbal drugs have recently been reviewed [166].

Therefore, considering the growing body of experimental evidence about the medicinal efficacy of herbs, it is necessary to identify their active principles responsible for such effects. This should be followed by studies to explore the mechanisms of action of active principles of interest. It is hoped that the progress in the refinement of our analytical tools will pave the way for the identification of more chemicals of therapeutic value from herbal and ethnomedicinal prescriptions. There is a hidden wealth of knowledge about naturally occurring medicinal compounds, which is yet to be explored in the years to come.

## Figures and Tables

**Table 6:** Anticancer activity of some potent antioxidant carotenoids (recent data).

Bioactive Molecules	Indian Ethnomedicinal Plant Source	Chemical Classification	Associated Condition	Cell Line Tested	Biological Approach (In Vitro/ In Vivo)	Mode of Action	References
α-carotene	Tulsi (*Ocimum tenuiflorum*)	Carotenes	Lung carcinoma	LLC	In vitro, In vivo	Inhibits Lewis lung carcinoma metastasis and suppresses lung metastasis	[152]
β-carotene	Gajara (*Daucus carota* subsp. *sativus)*	Carotenes	Gastric cancer	gastric epithelial cells	In vitro	β-catenin signaling and oncogene expression.	[153]
Lycopene	Devataruni (Rose hips-*Rosa canina* L.)	Carotenes	Esophageal cancer		In vivo	Pro-apoptosis	[154]
			Human cervical cancer	HeLa cells	In vitro	Inhibition of cell viability, upregulation of Bax expression, and downregulation of Bcl-2 expression	[155]
Lutein	Sthulapushpa- Marigold (*Tagetes erecta*) petals	Xanthophylls	Human cervical cancer	HeLa cells	In vitro	Induce apoptosis by increasing ROS production, interaction with mitochondrial factors, and upregulation of caspase-3-mediated pathway resulting in fragmentation of nuclei DNA	[156]
Zeaxanthin	Kumkuma (*Crocus sativus*)	Xanthophylls	Gastric cancer	AGS, KATO-3, MKN-45, MKN-28, NCI-N87, YCC-1, YCC-6, SUN-5, SUN-216, YCC-16, SUN-668, SUN-484	In vitro	Upregulating intracellular ROS levels, and regulating AKT, MAPK, NF-KB, and STAT3 signaling pathways	[157]
β-cryptoxanthin	Tulsi (*Ocimum tenuiflorum*)	Xanthophylls	Human cervical cancer	HeLa	In vitro	Activated nuclear condensation and disruption of the integrity of the mitochondrial membrane, upregulation of caspase-3, -7, and -9 mRNA, and increased activation of caspase-3 proteins leading to apoptosis and nuclei DNA damage	[158]
Fucoxanthin	Kelp (seaweed)	Xanthophylls	Brain and spinal cord cancer	U251-human-glioma-cell	In vitro	Apoptosis by triggering ROS-mediated oxidative damage and dysfunction of MAPKs and PI3K-AKT pathways	[159]
Fucoxanthinol	Kelp (seaweed)	Xanthophylls	Human cervical cancer	HeLa, HepG2, and Jurkat cells	In vitro	Induces apoptosis by cleavage of DNA, LDH release, activation of caspase-3, and decrease in cell count	[160]
β-ionone	Shatapattri (*Rosa Centifolia*)	Apocarotenoids/cyclic isoprenoid	Hepatocellular carcinoma		In vivo	Apoptogenic signal induction mediated by downregulation of Bcl-2 and upregulation of Bax, PPAR-γ, and FOXO-1 expressions	[161]
Crocetin	Kumkuma (*Crocus sativus*-flower) and Gandhraj (*Gardenia jasminoides*-fruits)	Apocarotenoids	Esophageal squamous cell carcinoma	KYSE-150	In vitro	Activated mitochondrial-mediated apoptosis pathway with an eventual disruption of MMP, increased levels of Bax and cleaved caspase-3, and decreased levels of Bcl-2	[162]
Crocin	Kumkuma (*Crocus sativus*-flower)	Apocarotenoids	Breast cancer	HCC70, HCC1806, HeLa and CCD1059sk	In vitro	Inhibited cell proliferation mainly by disrupting the microtubule network	[163]
Picrocrocin	Kumkuma (*Crocus sativus*-flower)	Apocarotenoids	Skin cancer	SK-MEL-2	In vitro	Targeting signaling pathway of JAK/STAT5, cell cycle arrest and mitochondrial assisted apoptosis	[164]
Bixin	Sinduri (*Bixa orellana* L.)	Apocarotenoids	Cutaneous melanoma	A2058	In vitro	ROS-mediated cytotoxicity	[165]
